
JOURNAL INFORMATION
==============================
NLM Title Abbreviation: ScientificWorldJournal
Journal ID: ScientificWorldJournal
NLM Journal ID: 101131163
Journal ID: TSWJ

The Scientific World Journal

ISSN: 2356-6140
EISSN: 1537-744X
Publisher: Wiley

ARTICLE INFORMATION
==============================
PMCID: PMC6084190
PMID: 30147564
DOI: 10.1100/tsw.2001.207
Article ID: 647670
Article version: 1
Subjects: Short Report

Evaluation of Ex-Vivo Gene Transfer of Fadd-Dn to Pancreatic Islets

Fenjves Elizabeth S. *
Ochoa Maria S.
Barney Thierry
Molano Damaris
Pileggi Antonello
Ricordi Camillo
Inverardi Luca
Diabetes Research Institute, University of Miami School of Medicine, P.O. Box 016960 Miami, FL 33101, USA
*Elizabeth S. Fenjves: efenjves@miami.edu
Publication date: 2001
Electronic publication date: 2001 Oct 26
Volume: 1
First page: 92
Last page: 92
Copyright: Copyright © 2001 Elizabeth S. Fenjves et al.
Copyright year: 2001
License: This is an open access article distributed under the Creative Commons Attribution License, which permits unrestricted use, distribution, and reproduction in any medium, provided the original work is properly cited.
License URL: https://creativecommons.org/licenses/by/3.0/

==============================
Miami *Nature Biotechnology* Short Reports
*TheScientificWorld* (2001) 1 (S3), 92SR
ISSN 1532-2246; DOI 10.1100/tsw.2001.207

### EVALUATION OF EX-VIVO GENE TRANSFER OF FADD-DN TO PANCREATIC ISLETS

Elizabeth S. Fenjves*, Maria S. Ochoa, Thierry Barney, Damaris Molano, Antonello Pileggi, Camillo Ricordi, Luca Inverardi

Diabetes Research Institute, University of Miami School of Medicine, P.O. Box 016960 Miami, FL 33101, USA
* Efenjves@miami.edu

**INTRODUCTION.** Type 1 diabetes is an autoimmune disease resulting in the destruction of the insulin-producing beta cells of the islets of Langerhans. The transplantation of healthy insulin-producing islets into patients with type 1 diabetes has been pursued for decades as an important treatment modality for this disease. Reproducible success of this approach has been complicated by early loss of transplanted cells due to apoptosis. The clinical utility of allogeneic islet transplantation will be tremendously enhanced if transplanted islets can be genetically engineered to evade pro-apoptotic stimuli at the engraftment site. Non-viral-based vectors such as lipofectants are good candidates to assess the role played by ex-vivo gene transfer of anti-apoptotic genes into primary islets. The aim of this study was to lipofect the dominant negative mutant of FADD (FADD-DN) into murine pancreatic islets to assay the effect of this transgene on islet viability.

**METHODS.** Murine pancreatic islets were isolated and lipofected with plasmids coding for FADD DN (NFD-4)(1) (kindly provided by Dr. G. Barber) or the reporter Green Fluorescent Protein (GFP). Both these genes were cloned into a pLXSN-based plasmid (2) directly downstream of the LTR. Various lipofectants and lipofection parameters were tested and optimal conditions were established for lipofection of islets at 12ug FUGENE-6 and 2-5ug of DNA 1000 islet equivalents. Islets were washed briefly with serum-free antibiotic-free DMEM followed by a 10-minute treatment with Hanks Disassociation Buffer (Gibco-BRL) at 24$^{o}$C to disrupt the islet capsule. Initial experiments were done with pLX-GFP-SN to determine transfection rates. Reporter gene expression was determined 48-72 hr after lipofection by trypsinizing islets into single cells and doing both flow cytometry and histological examination. Transfection efficiencies varied between 8% and 25% in islets. Islets transfected with either the reporter protein or FADD-DN were subjected to FAS induction using IFNγ and IL-1α. Apoptosis was induced using CD-95 (anti FAS) and protein-G or TNF-alpha. TUNEL assay was used to measure cell death due to apoptosis. To assay whether the insulin secretion levels of these cells were impaired, standard ELISA were performed and islets were transplanted into a syngeneic diabetic host to assay for diabetes reversal.

**RESULTS.** Islets that were transfected with either gene and not subjected to FAS induction or to pro-apoptotic assault showed apoptosis levels of 12-18%. Islets transfected with GFP and induced showed apoptosis levels of 75% compared to 25%-50% for islets transfected with FADD-DN and induced. Insulin secretion was not adversely affected by the transgene or the lipofection. Furthermore, the FADD-DN transfected islets reversed diabetes faster than the GFP-transfected islets.

**CONCLUSION.** Islet lipofection is both non-toxic and efficient. The possibility of using this gene transfer technique to make islets resistant to apoptosis presents an exciting therapeutic application in islet transplantation. Future directions will aim to engineer apoptosis-resistant islets that may be useful in the clinical setting.

**REFERENCES.**

1. Hu, S., Vincenz, C., Buller, M., and Dixit, V.M. (1997) J. Biol. Chem. 272, 9621-9624
2. Miller, A.D., Miller, D.G., Garcia, J.V., and Lynch, C.M. (1993) 217, 581-599
